# Crowding Effects of Polystyrene Nanoparticles on Lactate Dehydrogenase Activity in Hydra Attenuata

**DOI:** 10.3390/jox10010002

**Published:** 2020-09-16

**Authors:** Joelle Auclair, François Gagné

**Affiliations:** Aquatic Contaminant Research Division, Environment and Climate Change Canada, 105 McGill, Montreal, QC H2Y 2E7, Canada

**Keywords:** lactate dehydrogenase, polystyrene, nanoplastics, fractal dimension, *Hydra attenuata*

## Abstract

Plastics pervade our environment and potentially release important quantities of plastic nanoparticles (NPs) from degradation in the environment. The purpose of this study was to examine the crowding effects of polystyrene NPs on lactate dehydrogenase (LDH) in vitro and following exposure to *Hydra attenuata*. First, LDH activity was measured in vitro in the presence of filamentous (F-)actin and NPs (50 and 100 nm diameter) to determine changes in viscosity and the fractal kinetics of LDH. The fractal dimension (fD) was also determined using the rescaled range analysis procedure. Secondly, these changes were examined in hydra exposed to NPs for 96h to concentrations of NPs. The data revealed that the addition of F-actin increased the rate of LDH at low substrate (pyruvate) concentrations compared to LDH alone with a gradual decrease in the rate with the addition of pyruvate, which is characteristic of the fractal behavior of enzymes in crowded environments. The addition of 50 and 100 nm NPs also produced these changes, which suggest that NPs could change the space properties of the LDH reaction. The fD was reduced to 0.85 and 0.91 with 50 and 100 nm NPs compared to 1.093 with LDH alone. Decrease in the fD was related with increased amplitudes and frequency in viscosity waves in the reaction media. Exposure of hydra to NPs confirmed the increase in LDH activity and the fD was significantly correlated with LDH activity (r = −0.5). Correction of LDH activity (residuals) still revealed an increase in LDH activity in hydra suggesting increased anaerobic metabolism by NPs. In conclusion, the presence of NPs in the intracellular space decreased the fD, which could influence LDH activity in organisms exposed to NPs.

## 1. Introduction

The contamination of the aquatic environment by plastic waste represents one of the major pollution problems of the 21st century [1]. Plastic materials degrade in the environment forming smaller particles reaching the micro and nanoscales (<1000 nm) [2]. For example, one polystyrene coffee cup cover released over 150 million plastic nanoparticles (NPs) with a mean size of 200 nm after mixing for 58 days [3]. Thus, the degradation of plastic waste has the potential to release staggering amounts of NPs in the environment. The toxic properties of plastic NPs will differ from larger microplastic debris given their capacity to permeate not only tissues but cells as well [3]. The persistence and long-term effects of plastic NPs in cells are largely misunderstood at the present time. It is hypothesized that the mere presence of NPs in the cytoplasm could produce changes in the biophysical properties of the cytoplasm space domain where many biochemical pathways take place. From the nanoecotoxicity perspective, plastic NPs are interesting case to examine the crowding effects of the intracellular space given their chemical stability/“inertness”. Indeed, NP toxicity is not only initiated by the release of potentially toxic molecules, but by the size/shape, surface area properties (reactivity) and vector effects i.e., the transport of chemicals by NPs [4]. The distribution of plastic NPs in ecosystems is also largely unknown owing to the lack of methods to detect particles. Hence, plastic NPs could already contaminate our water bodies, pass biological barriers and find their way at the subcellular level [5].

The contamination of the intracellular space by plastic NPs could lead to increased crowding in the cytoplasm and introduce hydrophobic interaction. Indeed, polystyrene plastics are polymers of nonpolar styrene (vinyl benzene) repeats, thereby increasing not only molecular crowding but introducing important hydrophobicity from the high surface to volume ratio of NPs. The presence of NPs in hydra was detected using a solvatochromic dye (Nile red) which can detect changes in micropolarity [6]. The levels of uptake of plastic NPs using the Nile red methodology in Hydra exposed to translucent polystyrene NPs was statistically similar in Hydra exposed to fluorescently-labeled polystyrene NPs. The study revealed that exposure of hydra to polystyrene NPs was bioavailable and leads to increased lipid mobilization. It was proposed that the mobilization of lipids in cells could represent a means to remove and detoxify internalized plastic NPs. Moreover, the presence of NPs in tissues was associated to anisotropic changes normally associated with nonpolar liquid crystals [7]. The introduction of hydrophobic plastic NPs in cells could therefore influence the biophysical properties of the cytoplasm such as lipid mobilization, viscosity, protein fibrillation and the formation of anisotropic liquid crystals. In addition, NPs were shown to interact with proteins/lipids forming a corona at the surface, which could change the internal (space) organization in the cytoplasm [8,9]. Changes in the fractal space of the complex protein networks in the cytoplasm modulate enzyme kinetics in a characteristic way. Enzyme activity is usually determined by saturating concentrations of substrate in dilute enzyme solutions to determine the steady state reaction rate. However, enzyme kinetics behave differently in the crowded/fractal environment usually found in cells compared to diluted media. The cytoplasm is a complex environment of protein networks of fractal nature [10,11]. In these conditions, the rate of enzymes deviates from the normal parabolic behavior with substrate concentration, i.e., the reaction rates increase with the addition of substrate reaching a “plateau phase” or maximal velocity. In fractal environments, the rate increases more quickly (acceleration) first at low substrate concentration, which tends to decrease as the substrate concentration increases (deceleration), forming an inverted U-shaped pattern [12]. Another consequence of fractal environment is that the enzyme constants (Km or Vmax) are no-longer constant and become time-dependent [13,14]. The fractal property of enzyme reactions could be determined by the fractal dimension (fD) through the time course of product formation [13]. The term fractal indicates that the signal (enzyme rate) will occur at different scales following a power law of fractals: N α ε^−fD^ where N is the number (intensity) of the pattern or signal, ε the scale to the fD power. Hence, a decrease in fD could increase the rate of reaction. The fD is always smaller than the Euclidian dimension which can be taken as measure of the “complexity” of space. As the space becomes crowded, the fD will be lower than the normal Euclidian space in a power law way. In the present study, we selected the enzyme lactate dehydrogenase (LDH), which is known to interact in crowded (fractal) environments of F-actin networks [15]. LDH is a marker enzyme of anaerobic glycolysis when oxygen is limited and catalyzes the reaction: pyruvate + NADH ↔ lactate + NAD^+^. It is also used as a biomarker of anaerobic metabolism during low oxygen levels in tissues. Microplastics and plastics could clog tissues thereby limiting the diffusion and handling of oxygen in cells.

The purpose of the study was therefore to examine the fractal kinetics of LDH in crowded environments by the natural binding environment of F-actin and by polystyrene NPs of 50 and 100 nm diameter. This will determine if plastic NPs could change the fD of the LDH reaction and change LDH activity. The fractal behavior of the LDH reaction will be also determined in hydra exposed to polystyrene NPs to confirm whether NPs could change the fD of the LDH reaction. The null hypothesis consists of polystyrene NPs having no influence on the fD of the LDH reaction at low and saturating concentrations of the substrates.

## 2. Materials and Methods

### 2.1. In Vitro LDH Activity

The influence of polystyrene NPs on LDH activity was first examined in vitro with commercial preparations of LDH and F-actin to highlight changes in the spatial properties of the LDH reaction by NPs. This was followed by Hydra exposed to NPs to confirm whether the in vitro changes could be reproduced in exposed organisms. Lactate dehydrogenase (LDH) from pig muscle, 9-(dicyanovinyl) julolidine (DCVJ), reduced NADH, ATP and G-actin were purchased from Sigma Chemical company (Mississauga, Canada). The LDH assay was performed in 100 mM NaCl containing 5 mM KH_2_PO_4_, pH 7.2, 1 mM NaHCO_3_ and 10 µM DCVJ reaction media. The reaction was started with 1 mM NADH and increasing amounts of pyruvate (0.2, 0.5, 0.7, 1 and 1.5 mM) to determine the time dependent decrease in NADH. The disappearance of NADH was measured at 360 nm excitation and 450 nm emission each 30 s for 60 min in dark microplates using a microplate fluorescence reader (Flash mode, Synergy-4, Biotech Instruments, Winooski, VT, USA). Changes in viscosity were also determined by the DCVJ probe at 450 nm excitation and 520 nm emission during the LDH reaction [16]. The influence of F-actin and polystyrene NPs were also examined at 10, 20 and 30 µg/mL and 0.05 µg/mL for 50 and 100 nm polystyrene NPs in the reaction media, respectively (Polyscience, USA). Blanks consisted of the F-actin polymerization buffer alone (no ATP and G-actin) or water for NPs. The production of F-actin was generated overnight at 4 °C with 10 mg/mL G-actin in the presence of polymerization buffer: 10 mM Hepes-NaOH, pH 7.2, containing 100 mM KCl, 0.5 mM MgCl_2_, 1 mM CaCl_2_, and 0.2 mM ATP [17]. The formation of F-actin was measured at 450 nm before and after overnight (12 h) incubation.

### 2.2. Exposure of Hydra to Polystyrene Nanoparticles

Exposure of hydra to nanoplastics was performed as described previously [18,19]. Briefly, the hydra were cultured in media composed of 1 mM CaCl_2_ and 0.5 mM N-[Tris(hydroxymethyl)methyl]-2-aminoethanesulfonic acid (TES) buffer, pH 7.0 at 20 °C. They were fed on a daily basis with suspensions of *Artemia salina* brine shrimps. Transparent unlabeled polystyrene nanoplastics (NPs) of 50 and 100 nm diameter were purchased from Polyscience Inc. (USA) and were diluted to 1 mg/mL in MilliQ water. The particle size distribution was confirmed using Dynamic Light Scatter analysis (DLS; 532 nm laser Mobius Instrument, Wyatt Technologies, Santa Barbara, CA, USA) in the presence of MilliQ water and in the exposure media [18]. A concentration of 20 mg/L of 50 nm of NPs contained 0.52 × 10^12^ particles/L. Hydra were exposed to increasing concentrations of 50 nm NPs: 1.25, 5, 10, and 20 mg/L for 96 h at 20 °C. These concentrations were selected to produce morphological alterations in the hydra and were not usually found in the environment but could be reached during a spillage situation of industrial sources of plastics. At the end of the exposure period, hydra were washed in the culture media and the hydra were homogenized on ice using a Teflon pestle tissue grinder in 0.5 mL of 100 mM NaCl, 5 mM KH_2_PO_4_, pH 7.2, containing 0.1 µg/mL apoprotinin and 1 mM EDTA. The homogenate was allowed to decant in ice for 60 min to remove large debris.

LDH activity in hydra homogenates was determined at saturating conditions, i.e., 1 mM pyruvate and NADH or to 1 mM NADH with increasing concentrations of pyruvate (0.1–2 mM). The reaction mixture was allowed to incubate for 30 min and fluorescence readings were taken every 30 s using a microplate reader. Total proteins were determined by the Coomassie blue dye binding methodology [20]. Serum bovine albumin was used for calibration. The data were expressed as decreased fluorescence (relative fluorescence units)/min/mg proteins.

### 2.3. Data Analysis

The in vitro LDH experiments were repeated *n* = 3 times and the biochemical assays were run in duplicate. Hydra were exposed to increasing concentrations of NPs at *n* = 3 organisms per concentration and the data were examined for normality and homogeneity of variance using Shapiro–Wilk and Bartlett tests, respectively. The data were normally distributed with homogenous variance and analyzed using one-way (concentration) ANOVA followed by the Least Square Difference test as the post-hoc test. The calculation of the fractal dimension (fD) was based on the rescaled range analysis or the Hurst exponent procedure of the enzyme reaction (formation of product) in time [21]. The rescaled range analysis is based on the relationship: R/S = k (t)^H^, where R is the data range (Maximum–Minimum value), S the standard deviation, k a constant, t time and H the scaling exponent. The H exponent is related to fD by the following equation fD = 2 − H. The H exponent is related to long-term memory of time series. A value of H = 0.5 indicates random motion and values > 0.5 indicates a long-term positive auotocorrelation. The fD is related to the space filling capacity related to the signal or process. The star symbol * indicates significance from the proper controls (*p* < 0.05).

## 3. Results

The stability of polystyrene NPs was examined in the hydra media at 1 h and 96 h following in the hydra media by DLS analysis. The analysis revealed the mean diameter of hydra after 1 h dissolution (53 ± 0.5 nm) and after 96 h of dissolution (55.3 ± 0.5). The polydispersity index was significantly increased from 1.3 ± 0.8 to 4.6 ± 1.3 at 1 and 96 h, respectively. The Zeta potential remained stable during the 96 h exposure time in the hydra media at −1.48 ± 0.06 mvolts.

**Figure 1:**
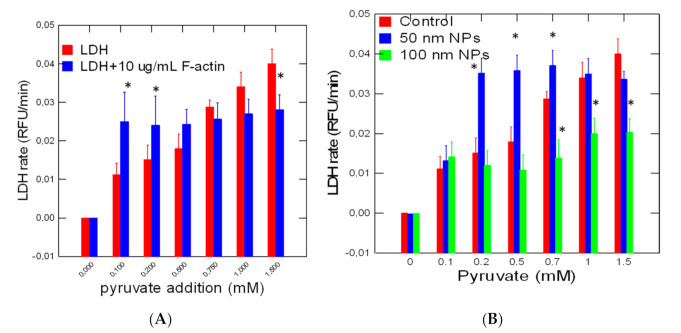
The fractal kinetic behavior of LDH in the presence of F-actin and 50 nm nanoparticles (NPs). LDH activity was determined alone and in the presence of F-actin (**A**) and 50/100 nm diameter NP (**B**). The data represent the mean with standard error for N = 3 replicates per treatment. The treatments were all significant from controls. The star symbol indicates difference between the crowding agents (F-actin, 50 and 100 nm NPs) and LDH alone. * *p* < 0.05.

**Figure 2:**
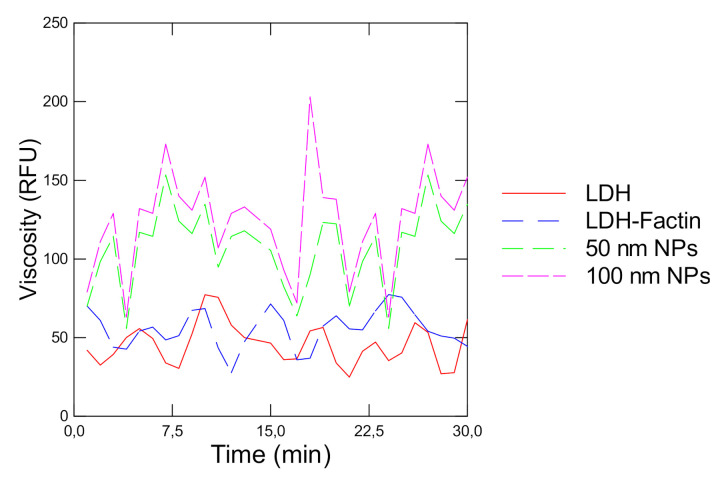
Change in viscosity by F-actin and NPs during the LDH reaction. The viscosity probe 9-(dicyanovinyl) julolidine (DCVJ) was added during the LDH reaction in the presence of 0.4 mM pyruvate. Fluorescence was measured at 450 nm excitation and 520 nm emission.

The activity of LDH in the presence of its natural protein network F-actin and polystyrene NPs was revealed in vitro (Figure 1A,B). The data showed that the reaction rate gradually increased (ANOVA F = 34, df = 6, *p* < 0 = 0.05) with the addition of pyruvate reaching saturation at circa 1 mM pyruvate. When polymerized F-actin was added to the reaction mixture, an increase (ANOVA F = 7, df = 6, *p* < 0 = 0.05) in the reaction rate was observed at low pyruvate concentration followed by a gradual decrease as the concentration increased compared with LDH alone. This pattern is characteristic of the fractal behavior of enzyme kinetics in crowded environments. The addition of polystyrene NPs also produced a similar pattern (ANOVA F = 34, df = 6, *p* < 0.05) but the effects started at 0.4 mM of added pyruvate compared to effects observed at 0.2 mM pyruvate with F-actin. This suggests that NPs can change LDH activity just by changing the fractal properties of space (fD) for LDH activity. During the LDH reaction, the viscosity probe DCVJ revealed an oscillatory behavior during the LDH reaction (Figure 2). Viscosity waves were observed during the LDH reaction alone with amplitudes in viscosity with a period of 5–6 min. In the presence of F-actin, the oscillatory changes in viscosity were similar but was somewhat dephased by 3 min relative to LDH alone. In the presence of 50 nm NPs, an increase in the amplitude of viscosity was observed with shorter periods of 3–4 min (higher frequency) which suggests amplitude and frequency modulation of viscosity by NPs during the LDH reaction. The Hurst exponent and the fD were also determined for the LDH reaction rate in the presence of F-actin and 50 nm NPs (Table 1). The analysis revealed that the Hurst exponent deviated further from random motion (H = 0.5) in the presence of F-actin and 50 and 100 nm polystyrene NPs. The H exponent of the LDH reaction alone was at 0.907 and significantly increased (ANOVA F = 11, df = 6, *p* < 0.05) to 1.01 in the presence of F-actin. The addition of 50 and 100 nm NPs in the reaction media also significantly increased (ANOVA F = 8.2, df = 6, *p* < 0.05) the H exponent with the 50 nm NPs followed by the 100 nm NPs at 0.05 µg/mL (ANOVA F = 7.2, df = 6, *p* < 0.05). This is consistent with the observation that on a particle basis, 50 nm NPs (0.13 × 10^11^ particles/mL) contained eight times more particles than the 100 nm NPs (0.17 × 10^10^ particles/mL) on a mass basis. The fD revealed that the dimension of the LDH reaction significantly decreased with the addition of F-actin, for 50 and 100 nm NPs suggesting that the space-filling capacity of the reaction media was reduced by these colloids and influenced the LDH reaction rate. The fD decreased the most with the 50 nm NPs (0.13 × 10^11^ particles/mL) compared to 100 nm NPs (0.17 × 10^10^ particles/mL) and F-actin.

**Figure 3:**
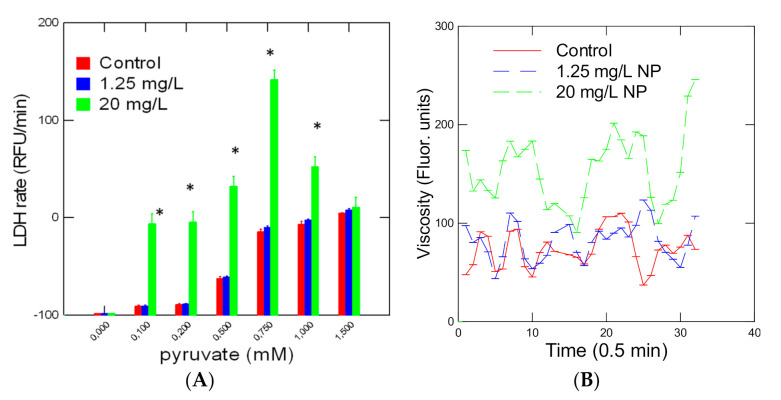
Rate change in LDH activity in hydra exposed to polystyrene NPs. LDH activity was determined with increasing amounts of pyruvate (**A**) and changes in viscosity (**B**). The data represent the mean with the standard error for N = 9 individuals per treatment. The star symbol represents significant difference compared to controls for the same pyruvate concentration. * *p* < 0.05.

**Figure 4:**
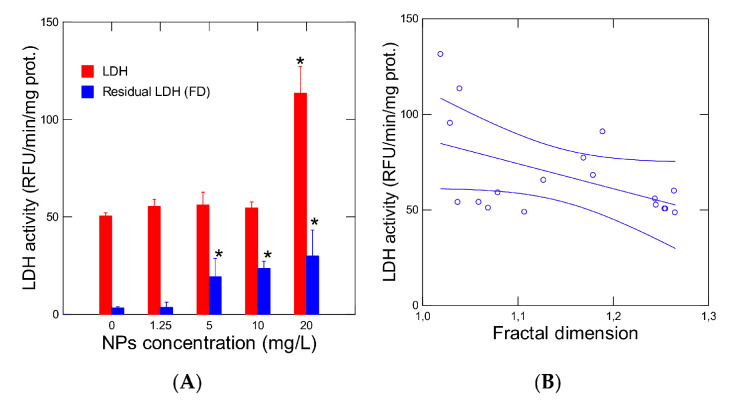
LDH activity and fractal dimension of LDH activity from Hydra exposed to NPs. Hydra were exposed to 50 nm NPs for 96 and analyzed for LDH activity (**A**) and the correlation with the fractal dimension (fD) (**B**). The data represents the mean with the standard error for N = 9 individuals per treatment. The star symbol * indicates significance from controls. * *p* < 0.05.

The activity of LDH was also studied in hydra exposed for 96 h to increasing concentrations of 50 nm NPs (Figure 3). LDH activity was increased (ANOVA F = 100, df = 6, *p* < 0.001) by the addition of pyruvate into the reaction media (Figure 3A). In hydra exposed to a low concentration of NPs (1.25 mg/L), no change in reaction rate was observed. In Hydra exposed to 20 mg/L NPs, a characteristic modulation of the reaction rate was observed i.e., increased reaction rates (ANOVA F = 121, df = 6, *p* < 0.001) at low concentrations followed by a decrease in the reaction rate at higher pyruvate concentrations. Viscosity changes were also observed in the incubation media at the low (0.2 mM) pyruvate concentration (Figure 3B). The changes in viscosity followed a periodic trend of 4–5 min in control organisms. The presence of the 1.25 mg/L NPs disrupted the pattern, showing decreased periodicity with some amplitudes increased. In hydra exposed to 20 mg/L, the amplitudes doubled with respect to controls and showed decreased periodicity at 2–3 min (higher frequency). Analysis of the fractal dimension of the LDH reaction revealed a significant decrease (ANOVA F = 4.7, df = 6, *p* < 0.05) in the fractal dimension in hydra exposed to NPs (Table 1). The fractal dimension of the LDH reaction in control Hydra was 1.255 and was closer to 1.5 corresponding to Brownian motion or white noise. Following exposure to NPs, the fractal dimension was reduced to 1.106 indicating more deviation from random (Brownian) motion and limited (fractalized) space. LDH activity was measured in Hydra at saturating amount of pyruvate of 1 mM was significantly elevated (ANOVA F = 55, df = 4, *p* < 0.01) at the highest NP concentration (Figure 4A). A significant correlation (r = −0.50; *p* < 0.05) was observed with the fractal dimension fD (Figure 4B). This suggests that decreasing the dimensionality of the LDH reaction was associated with increased activity in Hydra. The residual LDH activity was calculated and was included in Figure 4A. The analysis revealed that LDH activity was still significantly elevated at 5, 10 and 20 mg/L and was not solely due to decreased fD of the LDH microenvironment.

## 4. Discussion

This study examined the biophysical properties of polystyrene nanoparticles towards LDH activity. The influence of NPs was investigated initially in vitro to seek out changes in the spatial properties of the LDH reaction. The addition of NPs in the reaction media led to a significant decrease in the fD which was associated with increased activity in LDH activity in vitro. The increase in LDH activity could be the result of reduced dimension (fD) during the reaction. The reduced dimension was associated with increased amplitudes in viscosity waves. The increase activity in LDH in vitro and in exposed hydra was associated with increased amplitudes and frequency in viscosity during the reaction. A decrease in the fD from 1.09 to 0.85 was associated with a three-fold increase in amplitudes in viscosity indicating that reduced dimensionality could result in high changes in the biophysical properties of the reaction environment. This was also observed in hydra exposed to NPs where a 0.15 decrease in the fD was associated with a circa 2.5-fold increase in the amplitude in viscosity waves. The LDH enzyme complex oscillated between a relaxed and tensed state during its activity but at time scales of 260 µsec [22]. The bimolecular rate process occurring at different time scales (nsec–usec) indicates a complex dynamical structure of the substrates binding process. However, this time scale is well below the time scale on viscosity changes (min) suggesting that another macromolecular process was at play. It is possible that synchronized movement of substrates (channeling?) with the LDH enzymes was associated to the viscosity changes. Increased compactness of LDH by cationic polyehtyleneimine polymers was also shown in pig heart LDH [23]. This study showed that the nature of the interaction involved amine groups of polyethyleneimine polymers to the LDH enzyme in addition to increased hydrophobic interaction, which lowered the reaction rate of LDH. The decrease in turnover was enhanced with lower molecular weight polymers. In the case of polystyrene NPs, the decrease in LDH rate at saturating amounts of pyruvate involves the reduced fD of the enzyme’s environment, which could include a close interaction of the NPs with the enzyme. For the 50 and 100 nm NPs, decreased reaction rates were observed for high concentrations of pyruvate, which suggests a crowding effect of NPs in a fractal manner.

Exposure of hydra to polystyrene NPs for 96 h also led to increased activity in LDH and was correlated with fD. However, the residual LDH activity was still increased with exposure to NPs. This suggests that NPs increased anaerobic metabolism in these organism, which was not entirely explained by the reduced dimension of the reaction environment. Increased amplitudes and frequency of viscosity were also observed in hydra exposed to 50 nm NPs and perhaps a consequence of reduced fD as well. In a previous study, exposure to 0.7 mg/L microplastics (1–5 µm diameter) increased LDH activity in the muscle of the European sea bass [24]. Interestingly, the increase in LDH activity in muscles, an anoxic response, was associated with increased oxidative damage of lipids (lipid peroxidation) suggesting that microplastics could perturb the redox maintenance in organisms. Oxidative stress was recently reported in *Hydra attenuata* exposed to polystyrene NPs [18]. The presence of NPs in Hydra also leads to the formation of nonpolar liquid crystals, indicating a change in polarity in the cytoplasm could have occurred in addition to reduced fD. In another study in carp, a 28-day exposure to microplastic particles also increased LDH activity in the plasma [25]. However, increased LDH in the plasma could have resulted from muscle damage, which releases LDH in the circulatory system, and not from crowding effects. Interestingly, high temperature and microplastics increased anaerobic metabolism (LDH) in cichlid fish [26]. This could be of importance in the context of global warming where increased temperature events would occur concomitantly with NP contamination. However, the influence of temperature on the fractal spatial organization in cells is not well understood at present time and will require more research.

In conclusion, the presence of polystyrene NPs influenced the kinetic LDH in a fractal way i.e., increased activity at low substrate concentrations followed by decreased activity as the concentrations increase. This involved a reduction in the fD as determined by the Hurst exponent with increased amplitudes in viscosity in the reaction media, indicating the crowding effect of NPs. These effects were also observed when hydra were exposed to 50 nm NPs for 96 h. In addition to reduced fD of the LDH reaction, NPs could also increase anaerobic metabolism and oxidative stress in hydra.

## Figures and Tables

**Table 1:** Hurst exponent analysis of lactate dehydrogenase (LDH) activity. Hurst exponent H was derived from the relationship: R/S = k (n observation)^H^. The fractal dimension D was obtained by the following equation D = 2 − H. The star symbol * indicates significance from the proper controls. for N = 3 replicates per treatment.

Conditions	Hurst Exponent	Fractal Dimension D
In vitro LDH		
Without F-actin	0.91 ± 0.003	1.09 ± 0.01
With F-actin	1.01 ± 0.01 *	1.00 ± 0.01 *
NP50 nm	1.15 ± 0.01 *	0.85 ± 0.02 *
NP100 nm	1.09 ± 0.02 *	0.91 ± 0.02 *
Hydra		
Controls	0.74 ± 0.03	1.25 ± 0.05
1.25 mg/L NP	0.75 ± 0.03	1.25 ± 0.04
5 mg/L NP	0.82 ± 0.04 *	1.18 ± 0.06 *
10 mg/L NP	0.87 ± 0.04 *	1.13 ± 0.05 *
20 mg/L NP	0.93 ± 0.04 *	1.11 ± 0.05 *
